# Adapting the 4Ms Framework for American Indian and Alaska Native Communities

**DOI:** 10.1093/geroni/igaf122.3697

**Published:** 2025-12-31

**Authors:** Richard Marottoli, Bill Benson, Breana Dorame, Megan Dicken, Crystal Gwizdala, Barry Wu, Jennifer Ouellet

**Affiliations:** Yale University School of Medicine, New Haven, Connecticut, United States; International Association for Indigenous Aging, Silver Spring, Maryland, United States; International Association for Indigenous Aging, Silver Spring, Maryland, United States; International Association for Indigenous Aging, Silver Spring, Maryland, United States; Yale School of Medicine, New Haven, Connecticut, United States; Yale School of Medicine, New Haven, Connecticut, United States; Yale School of Medicine, New Haven, Connecticut, United States

## Abstract

The 4Ms (Matters Most, Medication, Mentation/Mind, Mobility) provide a valuable framework for older individuals, and their caregivers and clinicians, for consideration of factors influencing health, daily function, and well-being. Through an iterative process involving internal discussions and external listening sessions including tribal organizations, brain health advocates and experts, we sought to adapt the language and visual imagery associated with the 4Ms to be more relatable and relevant to American Indian and Alaska Native communities. The final version reflects the individual 4M components and also incorporates cultural elements: a tree at the center, roots representing traditional core values, branches representing the 4Ms, and leaves depicting the daily activities and expressions of the 4Ms. The resulting visual images and terms can be further modified and adapted to reflect local tribal geographic elements, language, and traditions.

